# Impacts of osteosarcopenia on musculoskeletal health, risks of falls and fractures, and activities of daily living among population aged 50 and above: an age- and sex-matched cross-sectional analysis

**DOI:** 10.1007/s40520-024-02902-8

**Published:** 2024-12-27

**Authors:** Bao Tu Thai Nguyen, Ashleigh Peng Lin, Wan-Wan Yang, Shun-Jen Cheng, Yi-Jie Kuo, Tan Thanh Nguyen, Yu-Pin Chen

**Affiliations:** 1 The International Ph.D. Program in Medicine, College of Medicine, Taipei Medical University, Taipei, Taiwan; 2 Department of Orthopedics, Faculty of Medicine, Can Tho University of Medicine and Pharmacy, Can Tho, Vietnam; 3 Department of Medical Education, Taipei Veterans General Hospital, Taipei, Taiwan; 4 Biomedical Innovation Center, Wan Fang Hospital, Taipei Medical University, Taipei, Taiwan; 5 Department of Orthopedics, Wan Fang Hospital, Taipei Medical University, Taipei, Taiwan; 6 Department of Orthopedics, School of Medicine, College of Medicine, Taipei Medical University, Taipei, Taiwan; 7 No. 111, Sec. 3, Xinglong Rd., Wenshan Dist, Taipei City, 116 Taiwan

**Keywords:** Osteoporosis, Sarcopenia, Osteosarcopenia, Activities of daily living, Risk

## Abstract

**Aims:**

We conducted this study to investigate the impact of muscle loss on musculoskeletal health, fall and fracture risks, and activities of daily living (ADL) in elderly patients with osteoporosis.

**Materials and methods:**

This age- and sex-matched cross-sectional study analyzed data from a medical center involving patients aged ≥ 50 from 2020 to 2022. The included participants were formed into three groups: 100 with osteoporosis only, 100 with osteosarcopenia, and 50 control individuals without osteoporosis and sarcopenia. We compared groups based on their baseline characteristics, bone and muscle health measurements, and the risks of falls and fractures using the STRATIFY scale and FRAX, respectively. Additionally, ADL was assessed using the Barthel Index. A multivariate analysis was performed to identify factors associated with declined ADL in osteosarcopenic patients.

**Results:**

The mean age was 76.17 years, and 82% were female. The osteosarcopenic group demonstrated poorer bone and muscle quality and quantity, with greater risks of major osteoporotic-related fractures, hip fractures, and falls, as well as significantly decreased ADL than other groups. When comparing sexes, females exhibited worse performance than males across groups. Slow gait speed and high STRATIFY score are independent predictors of declined ADL in osteosarcopenic patients.

**Conclusion:**

Sarcopenia exacerbates osteoporotic patients, particularly women, worsening bone deterioration, increasing fall and fracture risks, and significantly impairing daily activities. Enhancing walking speed and reducing fall risk can boost independence in individuals with osteosarcopenia. Early detection, proper management, and preventive measures are essential for mitigating these adverse outcomes in high-risk individuals.

## Introduction

With the increasing elderly population, age-related bone and muscle quantity and quality declines are more concerning [1]. Osteoporosis, characterized by the reduction of bone mass, increases the risk of fractures, leading decreased physical and social function, and reduced overall well-being [2, 3]. Similarly, sarcopenia, defined by the loss of muscle mass and strength, is thought to contribute to an increased risk of falls, ultimately resulting in physical disability and impaired activities of daily living (ADL) [4, 5]. In 2009, a new term, “osteosarcopenia,” was introduced to highlight the co-occurrence of osteoporosis and sarcopenia, leading to a novel focus within the context of geriatric health [6].

Osteosarcopenia dramatically contributes to increased falls and fracture risk, and compromised ADL relative to either condition alone is conflicting. Microscopically, the bone and muscle cross-talk via mechanical stimulation and biochemical signaling is well-understood [7, 8]. Reduced muscle mass and strength associated with sarcopenia attenuate bone formation and enhance bone resorption, increasing the risk of fractures. Inflammatory cytokines associated with osteoporosis inhibit myogenic differentiation, further exacerbating sarcopenic conditions [9].

Despite this preliminary association, clinically, there is incongruous evidence supporting a synergistic effect whereby osteosarcopenia confers greater fracture risk than osteoporosis alone [1]. However, studies on whether the potentially increased falls and fractures associated with osteosarcopenia translate into significantly impaired ADL compared to osteoporosis alone are lacking.

Meanwhile, understanding the potential relationship among osteosarcopenia, fall and fracture risk, and ADL is essential for identifying high-risk patients and providing optimal care. Thus, we conducted a matched-pair cross-sectional study to investigate performance differences between the control, osteoporosis-alone, and osteosarcopenic groups, who shared the same background of age and sex. We hypothesized that individuals with osteoporosis and sarcopenia might exhibit a declined ADL and a higher risk of fracture than those without sarcopenia. Furthermore, we identified potential factors that could predict impaired ADL among osteosarcopenic patients.

## Materials and methods

This cross-sectional study utilized data from bone health examinations conducted at the Osteoporosis and Sarcopenia Care Center in Wan Fang Hospital, Taipei, Taiwan. Participants included men and women aged 50 years and older. Exclusions were made for individuals with prior hip or spine surgeries, previous diagnoses of osteoporosis or sarcopenia, or existing musculoskeletal diseases. The study adhered to the Declaration of Helsinki and received approval from the Ethics Committee of Taipei Medical University on April 1, 2022 (TMU-JIRB N202203088). Written consent was obtained from all participants for their inclusion in the study and the publication of their data.

### Study design

We retrospectively reviewed medical records from 1275 participants who came to our center from 2020 to 2022. Our objective was to evaluate the effect of sarcopenia in individuals with osteoporosis by examining outcomes across three distinct groups: individuals with osteoporosis only, those with osteosarcopenia, and a healthy control group. To ensure a balanced comparison, we utilized a 1:1 matching process based on age and sex. Participants with both osteoporosis and sarcopenia were paired with those who had osteoporosis alone, forming two groups of 100 participants each. Additionally, a healthy control group of 50 individuals was selected to serve as a comparative reference for these groups.

### Osteoporosis, Sarcopenia, and osteosarcopenia detection

The bone mineral density (BMD) of the lumbar spine and bilateral femoral neck was assessed using Dual-Energy X-ray Absorptiometry (GE Healthcare, Madison, WI, USA), with osteoporosis confirmed if the T-score fell below − 2.5 as per World Health Organization criteria [2]. Sarcopenia was diagnosed based on criteria from the Asian Working Group for Sarcopenia, involving a decline in muscle strength determined by handgrip strength (< 28 kg for males or < 18 kg for females) or a decreased physical performance according to slow gait speed, with a combined reduced appendicular skeletal muscle mass measured via bioelectrical impedance analysis with a cut-off of 7.0 kg/m^2^ for males and 5.4 kg/m^2^ for females [10]. Patients having both osteoporosis and sarcopenia were diagnosed with osteosarcopenia.

### Data collection

All participants completed a self-report to retrieve their demographic and anthropometric data, clinical history, and health-related information. Age, sex, body height and weight, body mass index (BMI), alcohol consumption, smoker status, and corticosteroid medication use were collected by our nursing staff. Muscle strength was evaluated by measuring the handgrip strength using a Jamar hydraulic dynamometer (Sammons, Preston, USA). In this study, we used the 4-meter walking test to assess participants’ gait speed [11]. They were instructed to walk a marked 4-meter path at their usual pace, beginning from a standing position and continuing until they crossed the finish mark. Timing started when one foot crossed the starting mark and ended when one foot crossed the finish mark. Gait speed was calculated in meters per second (m/s), with a cut-off of 0.8 m/s used to define slow speed [12]. The fracture risk assessment tool (FRAX) was used to predict the 10-year likelihood of major osteoporosis-related fractures and hip fractures among participants [13]. The falling risk was evaluated using the St. Thomas’s Risk Assessment Tool in Falling Elderly Inpatients (STRATIFY) Instrument [14]. This tool comprises five binary questions regarding fall history, agitation, visual impairment, incontinence, and transfer and mobility abilities. Each “yes” response scores one point, while each “no” scores zero, resulting in a total STRATIFY score ranging from 0 to 5 points. A higher score indicates a greater risk of falls, with two and above signifying a high risk. Additionally, the number of falls in the past year was recorded. The ADL was evaluated using the Barthel Index, a common ranking scale to assess the level of functional independence of the patient [15].

### Outcome interests

The three groups were compared based on baseline characteristics. Additionally, the groups’ fracture and fall risks and ADL were assessed and analyzed by sex. Factors related to ADL among patients with osteosarcopenia were also examined.

### Statistical analysis

Statistical analysis was performed using Statistical Package for Social Sciences (SPSS - version 24.0 for Windows, IBM Corp.). Descriptive statistics were utilized to summarize categorical variables in frequencies and proportions, while continuous variables were presented as means ± standard deviations. The normality of the data distribution was assessed using the Shapiro-Wilk test. Comparative analyses for continuous variables between three groups were carried out using one-way analysis of variance (ANOVA) for normally distributed data, the Kruskal-Wallis ANOVA test for non-normally distributed data, and the Chi-square test for categorical variables. A following post hoc test was applied when the ANOVA test results indicated statistically significant. Univariate and multivariate linear regression analyses were performed to determine factors associated with the impairments in ADL among osteosarcopenic patients. The results were considered statistically significant with a two-tailed p-value less than 0.05.

## Results

There were 250 participants included in our study, with a mean age of 76.17 years old, and females (82%) were more than males (18%). There were 200 osteoporotic patients, including 100 patients with sarcopenia and 100 patients without sarcopenia. Those groups and a control group of 50 participants were compared to each other. Table 1 presents the data for the included participants. The osteosarcopenic group had a significantly lower BMD (T-score: -3.64) compared to the osteoporosis-alone group (T-score: -3.28) and the control group (T-score: -1.78). Patients with both osteoporosis and sarcopenia also experienced a notable reduction in muscle mass and physical performance relative to those without sarcopenia and to control participants. Osteosarcopenic patients had higher risks of major osteoporotic-related fractures (21.25% vs. 17.97% vs. 11.35%, respectively, *p* < 0.001) and hip fractures (11.35% vs. 9.07% vs. 3.78%, respectively, *p* < 0.001) than patients without sarcopenia and control ones. Significantly higher STRATIFY score (*p* = 0.013), incidence of high risk of falls (23%, *p = *0.007), and incidence of falls in the past year (42%, *p* = 0.048) were seen in patients with sarcopenia compared to other groups. The ADL declined traumatically as the Barthel Index was significantly lower in patients with osteosarcopenia compared to osteoporosis-alone and control participants (91.80 vs. 97.60 vs. 98.80, respectively, *p* < 0.001).

**Table 1:** Characteristics of all participants

Variable	Total (*n* = 250)	Control (*n* = 50)	Osteoporosis		*P* value
			No sarcopenia (*n* = 100)	Sarcopenia (*n* = 100)	
Age, y	76.17 ± 8.447	76.18 ± 5.442	76.17 ± 9.078	76.17 ± 9.078	1.000
Sex					
Female	205 (82)	41 (82)	82 (82)	82 (82)	1.000
Male	45 (18)	9 (18)	18 (18)	18 (18)	1.000
BMD					
T-score	-3.13 ± 1.032	-1.78 ± 0.535^bc^	-3.28 ± 0.767^ac^	-3.64 ± 0.863^ab^	< 0.001^*^
BMI, kg/m^2^	22.69 ± 3.858	25.63 ± 4.063^bc^	23.31 ± 3.386^ac^	20.60 ± 2.939^ab^	< 0.001^*^
Muscle mass, units	7.330 ± 1.386	7.798 ± 1.292^bc^	7.484 ± 1.415^ac^	6.942 ± 1.310^ab^	< 0.001^*^
Handgrip strength, kg	21.848 ± 5.989	24.592 ± 5.721^c^	23.681 ± 5.918^c^	18.643 ± 4.630^ab^	< 0.001^*^
Gait speed, m/s	0.790 ± 0.351	0.929 ± 0.237^c^	0.816 ± 0.329^c^	0.694 ± 0.392^ab^	< 0.001^*^
Alcohol consumption	0 (0)	0 (0)	0 (0)	0 (0)	
Current daily smoker	7 (2.8)	1 (2)	3 (3)	3 (3)	0.929
Corticosteroid use	7 (2.8)	0 (0)	0 (0)	7 (7)	0.005
Barthel index	95.52 ± 13.545	98.80 ± 6.512^c^	97.60 ± 8.804^c^	91.80 ± 18.416^ab^	0.001^*^
Fracture risk (FRAX),					
Major osteoporotic	17.96 ± 9.784	11.35 ± 4.911^bc^	17.97 ± 9.233^ac^	21.25 ± 10.518^ab^	< 0.001^*^
Hip fracture	8.92 ± 7.318	3.78 ± 3.130^bc^	9.07 ± 6.724^ac^	11.35 ± 8.081^ab^	< 0.001^*^
Falling risk					
STRATIFY score	0.67 ± 0.890	0.58 ± 0.906^c^	0.49 ± 0.659^c^	0.90 ± 1.030^ab^	0.013^*^
High risk (score ≥ 2)	38 (15.20)	8 (16.0)	7 (7.0)	23 (23.0)	0.007^*^
Falls within 1 year	83 (33.20)	15 (30.00)	26 (26.00)	42 (42.00)	0.048^*^

Data were presented under mean ± standard deviation/ n (%)

Abbreviations: BMD = Bone mineral density; BMI = Body mass index; FRAX = Fracture risk assessment tool; STRATIFY: St. Thomas’s Risk Assessment Tool in Falling Elderly Inpatients

^*^ Statistically significant

^a^*p* < 0.05 vs. control group

^b^*p* < 0.05 vs. no sarcopenia group

^c^*p* < 0.05 vs. sarcopenia group

Table 2 shows the participants’ performances by sex. In both males and females, patients having sarcopenia demonstrated the lowest BMDs than the other groups. In the male group, osteoporotic patients with sarcopenia exhibited a significant loss in muscle strength compared to those without sarcopenia and control ones (25.16 vs. 32.23 vs. 31.20, respectively, *p* < 0.001). Their fracture risk was higher than that of the control group but comparable to the osteoporosis-alone group. There was no significant difference in falling risk among the male patients when comparing their STRATIFY scores. Among females, those with osteosarcopenia had significantly lower bone and muscle quantity and quality, more decreased ADL, and more significant fall risk compared to the other two groups. Besides, females with osteosarcopenia exhibited higher risks of major osteoporosis-related fractures (22.79% vs. 14.22%), hip fractures (12.01% vs. 8.33%), and falls (0.93 vs. 0.78) than males.

**Table 2:** Characteristics of the participants by sex

Variable	Male (*n* = 45)	Control (*n* = 9)	Osteoporosis		*P* value	Female (*n* = 205)	Control (*n* = 41)	Osteoporosis		*P* value
			No sarcopenia (*n* = 18)	Sarcopenia (*n* = 18)				No sarcopenia (*n* = 82)	Sarcopenia (*n* = 82)	
Age, y	76.82 ± 9.649	82.33 ± 6.614	75.44 ± 10.007	75.44 ± 10.007	0.201	76.03 ± 8.179	74.83 ± 4.141	76.33 ± 8.919	76.33 ± 8.919	0.578
BMD										
T-score	-3.09 ± 1.019	-1.79 ± 0.408^bc^	-3.19 ± 0.821^a^	-3.63 ± 0.844^a^	< 0.001^*^	-3.13 ± 1.037	-1.78 ± 0.564^bc^	-3.30 ± 0.759^ac^	-3.64 ± 0.872^ab^	< 0.001^*^
BMI, kg/m^2^	22.70 ± 3.464	24.66 ± 1.287^c^	22.88 ± 3.821	21.55 ± 3.496^a^	0.01^*^	22.69 ± 3.948	25.84 ± 4.431^bc^	23.41 ± 3.301^ac^	20.39 ± 2.784^ab^	< 0.001^*^
Muscle mass, units	9.11 ± 1.045	9.63 ± 0.797	9.09 ± 1.066	8.87 ± 1.089	0.203	6.94 ± 1.121	7.40 ± 0.997^c^	7.13 ± 1.228^c^	6.52 ± 0.918^ab^	< 0.001*
Handgrip strength, kg	29.19 ± 5.996	31.20 ± 8.759^c^	32.23 ± 4.610^c^	25.16 ± 2.565^ab^	< 0.001*	20.24 ± 4.638	23.14 ± 3.573^c^	21.80 ± 4.307^c^	17.21 ± 3.649^ab^	< 0.001*
Gait speed, m/s	0.84 ± 0.363	0.81 ± 0.285	0.87 ± 0.295	0.83 ± 0.463	0.757	0.78 ± 0.348	0.96 ± 0.221^bc^	0.80 ± 0.337^bc^	0.66 ± 0.371^ab^	< 0.001*
Current daily smoker	3 (6.667)	0 (0)	2 (11.111)	1 (5.556)	0.535	4 (1.951)	1 (2.439)	1 (1.220)	2 (2.439)	0.826
Corticosteroid use	4 (8.889)	0 (0)	0 (0)	4 (22.222)	0.037	3 (1.463)	0 (0)	0 (0)	3 (3.659)	0.102
Barthel index	97.11 ± 11.798	99.44 ± 1.667	99.72 ± 1.179	93.33 ± 18.231	0.274	95.17 ± 13.901	98.66 ± 7.161^c^	97.13 ± 9.656^c^	91.46 ± 18.551^ab^	0.004^*^
Fracture risk (FRAX)										
Major osteoporotic	12.05 ± 6.316	7.58 ± 3.337^bc^	12.11 ± 6.561^a^	14.22 ± 6.274^a^	0.002^*^	19.25 ± 9.942	12.18 ± 4.837^bc^	19.26 ± 9.265^ac^	22.79 ± 10.658^ab^	< 0.001^*^
Hip fracture	7.05 ± 5.233	3.467 ± 2.889^bc^	7.57 ± 6.553^a^	8.33 ± 3.896^a^	0.001^*^	9.34 ± 7.649	3.85 ± 3.210^bc^	9.40 ± 6.756^ac^	12.01 ± 8.612^ab^	< 0.001^*^
Falling risk										
STRATIFY score	0.69 ± 0.874	1.11 ± 1.269	0.39 ± 0.502	0.78 ± 0.878	0.265	0.67 ± 0.895	0.46 ± 0.778^c^	0.51 ± 0.689^c^	0.93 ± 1.063^ab^	0.012^*^
High risk (score ≥ 2)	6 (13.33)	3 (33.33)	0 (0)	3 (16.67)	0.048^*^	32 (15.61)	5 (12.20)	7 (8.54)	20 (24.39)	0.016^*^
Falls within 1 year	13 (28.889)	4 (44.444)	3 (16.667)	6 (33.333)	0.281	70 (34.15)	11 (26.83)	23 (28.05)	36 (43.90)	0.055

Data were presented under mean ± standard deviation/ n (%)

Abbreviations: BMD = Bone mineral density; BMI = Body mass index; FRAX = Fracture risk assessment tool; STRATIFY: St. Thomas’s Risk Assessment Tool in Falling Elderly Inpatients

^*^ Statistically significant

^a^*p* < 0.05 vs. control group

^b^*p* < 0.05 vs. no sarcopenia group

^c^*p* < 0.05 vs. sarcopenia group

We performed univariate and multivariate regression analysis to identify the predictors of ADL in osteosarcopenic patients, as demonstrated in Table 3. The findings indicate that slow gait speed (β = 0.213, *p* = 0.001) and increased STRATIFY score (β = -0.375 *p* < 0.001) can independently predict declined ADL in those patients.

**Table 3:** Univariate and multivariate analyses for predicting activities of daily living among osteosarcopenic patients

Predictors	Activities of daily living			
	Univariate		Multivariate	
	β	*p*-value	β	*p*-value
BMD (T-score)	0.121	0.231		
BMI	-0.052	0.606		
Handgrip strength	0.242	0.015*	0.106	0.079
Gait speed	0.314	0.001*	0.213	0.001*
Muscle mass	0.100	0.323		
STRATIFY score	-0.471	< 0.001*	-0.375	< 0.001*
FRAX				
Major osteoporosis	-0.140	0.165		
Hip fracture	-0.132	0.190		

Abbreviations: BMD = Bone mineral density; BMI = Body mass index; FRAX = Fracture risk assessment tool; STRATIFY: St. Thomas’s Risk Assessment Tool in Falling Elderly Inpatients; *: Statistically significant

## Discussions

Our study highlighted that osteosarcopenic patients experienced reduced bone and muscle mass alongside an increased risk of falls and fractures. Additionally, these patients exhibited a more pronounced decline in ADL compared to those without sarcopenia and control participants. Female patients with osteosarcopenia demonstrated poorer performance than their male counterparts when compared to those with osteoporosis alone and control subjects. Gait speed and STRATIFY score emerged as independent predictors of ADL in osteosarcopenic patients.

The interaction between bone and muscle has been documented in numerous studies [7, 8, 16]. Research indicates that the loss of muscle may contribute to the loss of bone. Verschueren et al. found that sarcopenia led to a lower BMD and osteoporosis in men [17]. Similarly, Qin et al. reported a positive correlation between appendicular skeletal muscle mass index and lumbar BMD in the general population [18]. In our study, among the osteoporotic group, the presence of sarcopenia significantly reduced BMD than those without sarcopenia, aligning with prior findings. This underscores the importance of monitoring sarcopenia in osteoporotic patients, as the concurrent loss of muscle mass can further deteriorate bone health.

Sarcopenia significantly increases the incidence of falls and fractures in elderly people [19, 20]. A decrease in T-score contributes to an increase in fracture risk [21]. Since our osteoporotic patients having sarcopenia had a lower BMD, as mentioned above, they consequently faced a higher fracture risk compared to those without sarcopenia. Furthermore, females were more vulnerable to fracture since their risk was higher than that of males. While many health-related factors could contribute to a higher risk of fracture, the primary reason sarcopenia leads to fractures is likely due to poor bone quality and an increased likelihood of falls. Falls have been shown to result due to a decrease in muscle mass, strength, physical condition, and balance [22–24]. Among our participants, those with osteoporosis and sarcopenia had a higher fall score and a more fall accident in the past year than those without sarcopenia, with females at a greater fall risk than males. This finding might conflict with a previous study, which reported that men were more at risk of falls and fractures than women in a meta-analysis [25]. At any rate, falls are hazardous for the elderly as they can lead to fractures, disability, and even mortality [26]. Although falls have multiple causes, sarcopenia is a crucial factor in heightening fall risk [19, 27]. Therefore, protecting elderly people from falls is an essential strategy to prevent osteoporosis-related fractures.

The ADL reflects an individual’s ability to perform self-care tasks such as eating, dressing, or bathing [28]. The disability in ADL can result in poorer quality of life, potential institutionalization, and a significant economic burden on the healthcare system [29–31]. Sarcopenia is linked with a high risk of disability and reduced physical performance [32–34]. Our findings supported this, as the Barthel Index scores for patients with sarcopenia were notably lower than those without it. Furthermore, gait speed and STRATIFY score were associated with functional declines. This highlights the importance of addressing the presence of sarcopenia, as they are at a higher risk for ADL disability, necessitating appropriate management to enhance their physical status.

According to our results, sarcopenia is a serious concern for elderly patients as it impacts bone health, leading to a higher risk of fractures. Moreover, sarcopenia raises the chances of falls, further increasing the fracture risk. Women with sarcopenia are especially at risk, with higher incidences of both fractures and falls compared to men. It also reduces ADL, potentially leading to a poorer quality of life and a greater burden on healthcare providers [24, 30]. However, on a positive note, sarcopenia can be modifiable. Resistance exercise and proper nutrition have been shown to be beneficial in preventing and managing sarcopenia [35–37]. We suggest assessing gait speed or determining fall risk could help identify patients with osteosarcopenia at risk for reduced independence. The predictable value of walking speed and falls for worsened ADL was also reported in previous studies [38–40]. Therefore, early detection and intervention to improve walking speed and decrease the risk of falls might help them maintain their ability to care for themselves.

Several limitations should be noted in this study. Firstly, the cross-sectional design precludes establishing causal relationships between sarcopenia and changes in bone and muscle, as well as on ADL in osteoporotic patients. Secondly, participant comorbidities were not examined, which may have influenced their performances. Thirdly, the predominance of female participants, though unavoidable, may introduce bias due to the underrepresentation of males. Lastly, since our patients were recalled from a single medical center, the generalizability of the findings to broader populations is limited. To better generalize the effects of sarcopenia on osteoporotic patients, a multi-institutional or nationwide database study is recommended.

To our knowledge, this is the first matched-pair study to compare patients with osteoporosis and sarcopenia, those with only osteoporosis, and healthy participants. Our findings revealed that patients with osteosarcopenia had poorer performance outcomes. It is essential to pay more attention to sarcopenia, not only to enhance bone quality but also to reduce the risks of falls and fractures and improve the basic functional status of these patients.

## Conclusion

Sarcopenia worsens osteoporotic patients, particularly women, as it exacerbates bone tissue deterioration, leading to increased risks of falls and fractures, and also significantly impairs daily activities. Enhancing walking speed and reducing fall risk can boost independence in individuals with osteosarcopenia. Early detection, proper management, and preventive measures are essential for these high-risk individuals to mitigate these adverse outcomes.

## Data Availability

The data that support the findings of this study are available on request from the corresponding author.
